# A prospective cohort study of biomarkers of prenatal tobacco smoke exposure: the correlation between serum and meconium and their association with infant birth weight

**DOI:** 10.1186/1476-069X-9-53

**Published:** 2010-08-27

**Authors:** Joe M Braun, Julie L Daniels, Charles Poole, Andrew F Olshan, Richard Hornung, John T Bernert, Yang Xia, Cynthia Bearer, Dana Boyd Barr, Bruce P Lanphear

**Affiliations:** 1 Department of Epidemiology, University of North Carolina-Chapel Hill, Chapel Hill, NC, 27599-7435, USA; 2 Department of Pediatrics, Division of General and Community Pediatrics, Cincinnati Children's Hospital Medical Center, Cincinnati, OH 45229, USA; 3 Division of Laboratory Sciences, National Center for Environmental Health, Centers for Disease Control and Prevention, Atlanta, GA 30341, USA; 4 Department of Pediatrics, University of Maryland School of Medicine, Baltimore, MD 21201, USA; 5 Rollins School of Public Health, Emory University, Atlanta, GA 30322, USA; 6 Child & Family Research Institute, British Columbia Children's Hospital and the Faculty of Health Sciences, Simon Fraser University, Vancouver, British Columbia, CA

## Abstract

**Background:**

The evaluation of infant meconium as a cumulative matrix of prenatal toxicant exposure requires comparison to established biomarkers of prenatal exposure.

**Methods:**

We calculated the frequency of detection and concentration of tobacco smoke metabolites measured in meconium (nicotine, cotinine, and trans-3'-hydroxycotinine concentrations) and three serial serum cotinine concentrations taken during the latter two-thirds of pregnancy among 337 mother-infant dyads. We estimated the duration and intensity of prenatal tobacco smoke exposure using serial serum cotinine concentrations and calculated geometric mean meconium tobacco smoke metabolite concentrations according to prenatal exposure. We also compared the estimated associations between these prenatal biomarkers and infant birth weight using linear regression.

**Results:**

We detected nicotine (80%), cotinine (69%), and trans-3'-hydroxycotinine (57%) in most meconium samples. Meconium tobacco smoke metabolite concentrations were positively associated with serum cotinine concentrations and increased with the number of serum cotinine measurements consistent with secondhand or active tobacco smoke exposure. Like serum cotinine, meconium tobacco smoke metabolites were inversely associated with birth weight.

**Conclusions:**

Meconium is a useful biological matrix for measuring prenatal tobacco smoke exposure and could be used in epidemiological studies that enroll women and infants at birth. Meconium holds promise as a biological matrix for measuring the intensity and duration of environmental toxicant exposure and future studies should validate the utility of meconium using other environmental toxicants.

## Introduction

Fetal meconium is formed beginning in the 13^th ^week of gestation from swallowed amniotic fluid, shed epithelial cells, and intestinal secretions [1]. Meconium may be metabolically inert and concentrations of drugs and other toxicants are thought to represent cumulative gestational exposure over the latter two-thirds of pregnancy [1]. Ostrea and colleagues have reported that meconium is a sensitive matrix for measuring markers of gestational drug use and prenatal pesticide exposure [2-6].

Prenatal active and secondhand tobacco smoke exposure is a prevalent environmental exposure that is associated with adverse infant and childhood health outcomes [7,8]. Several studies have detected and quantified tobacco smoke metabolites including nicotine (NIC), cotinine (COT), and trans-3'-hydroxycotinine (3HC) in infant meconium samples [3,9-12]. In adult humans, 70 to 80% of inhaled nicotine (NIC) from tobacco smoke is metabolized into cotinine (COT) and 50% of COT is metabolized into trans-3'-hydroxycotinine (3HC) [13]. Comparable percentages of nicotine and other tobacco smoke constituents can cross the placenta and have been measured in the fetus [14].

Results from prior studies indicate that meconium NIC, COT, and 3HC concentrations are correlated with maternal report of tobacco smoke exposure. However it is unclear if meconium tobacco smoke metabolites reflect the intensity and duration of prenatal tobacco smoke exposure since prior studies have used self-report or biomarkers of tobacco smoke exposure at birth to represent gestational exposures [3,11,15]. Two studies have attempted to determine if meconium tobacco smoke metabolites are reflective of prenatal secondhand tobacco smoke (SHS) exposures and both have been limited by small sample sizes [11,15]

Two studies have examined the association between meconium tobacco smoke metabolite concentrations and childhood health outcomes [12,16]. Gray and colleagues reported decrements in birth weight, length, and head circumference among infants with detectable meconium tobacco smoke metabolite concentrations. Nuesslein et al. found a positive association between meconium cotinine concentrations and risk of respiratory infections in the first year of life. Both studies were relatively small and did not have other biomarkers of tobacco smoke exposure that allowed for comparison to meconium results.

Given the interest in using meconium as a biological matrix to measure prenatal toxicant exposure in epidemiological studies like the National Children's Study [17], the utility of meconium should be validated in reference to well established biomarkers of gestational exposure. We examined the utility of meconium as a biological matrix of prenatal tobacco smoke exposure using two approaches in the Health Outcomes and Measures of the Environment (HOME) Study. First, we examined the association between meconium tobacco smoke metabolites and serial self-report and serum cotinine biomarkers of prenatal tobacco smoke exposure. Second, we evaluated whether the two biomarkers (meconium tobacco smoke metabolites and serum cotinine) would produce similar results when investigating the association between prenatal tobacco smoke exposure and infant birth weight.

## Methods

### Study Sample

We used data collected from mothers and their infants participating in the HOME Study, an ongoing prospective birth cohort in the Cincinnati metropolitan area designed to examine low-level environmental toxicant exposure and the efficacy of injury and lead hazard controls in the home. From March of 2003 to January 2006, women were identified from seven prenatal clinics associated with three hospitals. Eligibility criteria for the study included: <19 weeks gestation; > 18 years old; living in a house built before 1978; living in Brown, Butler, Clermont, Hamilton, or Warren counties, intention to continue prenatal care and deliver at collaborating obstetric practices, negative HIV status; and not receiving seizure, thyroid, or chemotherapy/radiation medications. We mailed letters to 5,512 women > 18 years of age who were living in a home built before 1978 to see if they were eligible and interested in participating in our study. Of the 1,263 eligible women, 468 enrolled in our study. Our analyses were restricted to singleton infants.

### Tobacco Smoke Measurements

#### Self-Reported Exposure

Women self-reported secondhand and active tobacco smoke exposures for the periods between conception and 20 weeks (measured at 20 week home visit) and 20 weeks and birth (measured at a 4 week postpartum visit). Trained interviewers asked the women the average number of cigarettes they smoked per day, the number of smokers living in the home, and the number of cigarettes smoked per day by other people in the home for each time period. We classified the woman's exposure status during each time period as unexposed, exposed to secondhand tobacco smoke, and exposed to active tobacco smoke.

#### Serum and Meconium Biomarkers of Exposure

Women provided serum samples at 16 weeks gestation, 26 weeks gestation, and within 24 hours of birth. All samples were stored at -20°C until they were transported to the Centers for Disease Control and Prevention (CDC) laboratory for analysis, where they were stored at < -20°C. Serum samples were analyzed for cotinine, a biomarker of nicotine exposure, using high performance liquid chromatography-tandem mass spectrometry (HPLC-MS/MS) [18,19]. The limit of detection (LOD) for this assay was 0.015 ng/mL with a coefficient of variation (CV) ranging from 3-4% at high concentrations (1 ng/mL) to 10% at low concentrations (0.1 ng/mL).

Meconium specimens were collected from infants during their hospital stay by placing cellulose fiber inserts inside infant diapers. After it was soiled, the diaper and insert were initially stored in a labeled plastic bag in a designated hospital refrigerator until they were collected by study staff, usually within 24 hours. Meconium stools were collected throughout the hospital stay, until the first milk stool appeared. Study staff pooled meconium samples from diapers into polyethylene containers using a spatula. Pooled meconium samples were stored at -20°C until transported to CDC laboratories where they were stored at < -20°C. Study staff took care not to collect fibers from the diaper or cellulose insert.

For this analysis approximately 0.5 g of meconium were digested at room temperature in 3 mL of 5 N potassium hydroxide containing the internal standards (trideuterated nicotine, cotinine and hydroxycotinine; 1.25 ng of each per sample except for nicotine which was 5 ng). The digested material was extracted with methylene chloride and ethanol, back-extracted into hydrochloric acid, neutralized, buffered, and applied to a CleanScreen DAU column which was processed essentially as described by Ostrea et al [4]. HPLC-MS/MS was used to quantify the concentrations of the NIC, COT, and 3HC relative to the deuterated internal standards. Samples were analyzed using multiple reaction monitoring and concentrations were calculated from the ratios of native and labeled ions in the samples compared to a 10-point calibration curve incorporating the three analytes. Transition ions monitored in this work were 192.9/80.0 and 192.9/133.9 for hydroxycotinine, 177/98.1 and 177/80.1 for cotinine, and 162.9/130, 162.9/117 for nicotine. Corresponding single transitions were monitored for each internal standard. The LODs for these three compounds were 0.946 ng/g for NIC, 0.070 ng/g for COT, and 0.092 ng/g for 3HC.

Each analytical run included two blank samples and a low and high concentration quality control (QC) sample. The low and high QC materials were prepared from two separate, pooled samples of meconium that had been pre-tested and confirmed to have different levels of all three metabolites present. Each pool was prepared by taking 25 g of its respective meconium sample and diluting with 600 mL 5 N KOH. This was allowed to stir for 2 hr to dissolve the solid and assure even mixing. Each pool was dispensed with continuous mixing in 3 mL aliquots into 200 sequentially numbered 16 × 125 mm screw cap tubes. After all the tubes were prepared, they were stored at -70°C until use. The low-concentration QC samples had a relative standard deviation (RSD) of 25% (NIC), 8% (COT), and 14% (3HC), while the high-concentration QC samples had a CV of 15% (NIC), 5% (COT), and 6% (3HC). Accuracy evaluations were conducted at target concentrations of 0.5, 2.5 and 7.5 ng/g (2, 10 and 30 ng/g for nicotine). All samples had an analytical bias of less than ± 10%.

### Infant Birth Weight and Covariates

Infant birth weight (in grams) was abstracted from hospital medical records and was analyzed as a continuous variable. Maternal age, race, education, and marital status were gathered at the first prenatal care visit. Maternal depression was assessed using the Beck Depression Inventory-II (BDI-II) which was administered during a home visit at approximately 20 weeks [20]. Parity was obtained from maternal medical records. Maternal weight (in kg) was collected at the initial clinic visit at 16 weeks gestation.

### Statistical Analysis

We conducted our statistical analysis in two stages. First we examined the relationship between the various self-report and biomarkers of prenatal tobacco smoke exposure. Second, we examined and compared the association between meconium and serum biomarkers of tobacco smoke exposure and infant birth weight.

We started our statistical analysis by comparing women and infants with complete meconium, self-report, serum, and birth weight data to women with missing data. We corrected for the right-skewed distribution of serum cotinine and meconium tobacco smoke metabolite concentrations using the log_10_-transformation in analyses using continuous variables. Serum and meconium cotinine values <LOD were randomly imputed from the left tail of the log_10_-normal distribution [21].

#### Relationship between Self-Report and Biomarkers of Tobacco Smoke Exposure

We compared methods of classifying tobacco smoke exposures among mother-infant pairs with at least two prenatal serum cotinine measurements and a valid meconium measurement available. We created several measures of cumulative prenatal tobacco smoke exposure using either self-reported tobacco smoke exposure or prenatal serum cotinine concentrations.

First, we created a summary variable of self-reported prenatal tobacco smoke exposure over the course of pregnancy. This five category variable reflected the level and duration of exposure: unexposed, SHS exposure in one period, SHS exposure in both periods, active exposure in one period (the other period was unexposed or SHS exposure), and active exposure in both periods.

Next, we averaged the available serum cotinine measurements to create a continuous summary measure women's prenatal tobacco smoke exposure between 16 weeks gestation and birth. From this, we created categories of unexposed (< LOD), secondhand exposure (LOD to 3 ng/mL), and active exposure (> 3 ng/mL). The threshold of 3 ng/mL for active smoking was chosen based on results from the 1999-2004 National Health and Nutrition Examination Survey which compared self-reported smoking status and serum cotinine levels among a representative sample of the US population [22].

Among women with all three serum cotinine and meconium measurements, we further quantified cumulative exposure to prenatal tobacco smoke by creating a summary variable that described the number of prenatal serum measurements that a woman was exposed to secondhand or active tobacco smoke. This seven category, ordinal variable counted the number of measurements that a woman had serum cotinine concentrations indicative of secondhand (zero, one, two, or three) or active (one, two, or three) tobacco smoke exposure. Women in any of the secondhand categories could not have had active exposure at another time point, while women in the active categories could have had another serum measurement consistent with secondhand or no exposure.

Finally, we investigated whether the timing of the serum measurements influenced the association between the number of serum cotinine measurements indicative of secondhand or active tobacco smoke exposure and meconium NIC concentrations. We did this using a seven category variable that summarized the number and timing of serum cotinine concentrations consistent with secondhand and active tobacco smoke exposure at 16 weeks and birth. We limited this analysis to NIC concentrations because NIC was detected most frequently and the results with NIC concentrations were similar to those using COT and 3HC concentrations.

We calculated the geometric mean (GM) and corresponding 95% confidence interval (CI) of meconium tobacco smoke metabolite concentrations according to self-reported and serum cotinine concentration categories described above. We also calculated the proportion of infants with detectable meconium tobacco smoke metabolite concentrations according to self-reported or serum categories.

#### Association between Biomarkers of Prenatal Tobacco Smoke Exposure and Infant Birth Weight

The second stage of our analysis examined the association between biomarkers of tobacco smoke exposure and infant birth weight. We chose to examine birth weight because there is a well-established inverse relationship between serum cotinine concentrations and infant birth weight [23-28].

We compared the results for the different biomarkers of tobacco smoke exposure since many cohorts only have the resources to collect one exposure measurement. We estimated and compared the associations between continuous log_10_-transformed prenatal serum cotinine and meconium tobacco smoke metabolite concentrations and infant birth weight using linear regression. Coefficients from these analyses represent the mean change in infant birth outcome for a 10-fold increase in tobacco smoke biomarker concentration. In addition, we examined the association between categorical serum and meconium tobacco smoke metabolite concentrations and infant birth weight. Serum cotinine concentrations were categorized using the thresholds described above. Several different meconium tobacco smoke metabolite concentrations were used to discriminate secondhand from active tobacco smoke exposure based on sensitivity and specificity analyses.

In all of the analyses examining the association between prenatal tobacco smoke exposure and infant birth weight, we adjusted for confounders identified using a directed acyclic graph (DAG) [29]. DAGs are a better method to assess the role of confounding variables compared to change in estimate and significance testing approaches [30]. Based on our DAG, all models included maternal age, maternal education, maternal race, marital status, depression (), and maternal weight (kg). We did not adjust for gestational age since it was an intermediary on the causal pathway between prenatal tobacco smoke exposure and infant birth outcomes.

### Ethical Considerations

The Institutional Review Boards (IRB) of the University of North Carolina-Chapel Hill, Cincinnati Children's Hospital and Medical Center, and CDC approved this study. The Cincinnati Children's Hospital and Medical Center IRB was involved in the oversight of this study. All mothers provided written informed consent for themselves and their children prior to enrollment in the study.

## Results

Of the 468 women who enrolled in our study, 67 dropped out before delivery. We excluded 9 children from sets of twins and 3 still-born infants, leaving 389 infants. A total of 326 women and infants (83.8%) had at least two prenatal serum measurements and a meconium measurement available, many (n = 284, 73.0%) had all three prenatal serum measurements and a meconium measurement. Complete self-reported tobacco smoke exposure and meconium data was available for 316 (81.2%) women. Women with complete data were better educated, non-Hispanic white, married, and 25-34 years of age compared to women with incomplete data (Table 1).

**Table 1:** Distribution of demographic variables among mothers/infants in HOME study

Variable	All Women N = 389 (%)	Women with All Data N = 315 (%) *	Women Missing Any Data N = 74 (%)
**Maternal Race**			
White	237 (61.7)	209 (66.4)	28 (40.6)
Black	121 (31.5)	86 (27.3)	35 (50.7)
Other	26 (6.8)	20 (6.3)	6 (8.7)
Missing	5	0	5
**Maternal Education (years)**			
< 12	41 (10.7)	23 (7.3)	18 (26.1)
12	54 (13.8)	41 (13.0)	13 (18.8)
> 12	289 (74.5)	251 (79.7)	38 (55.1)
Missing	5	0	5
**Marital Status**			
Married	248 (64.6)	217 (68.9)	31 (44.9)
Single	136 (35.4)	98 (31.1)	38 (55.1)
Missing	5	0	5
**Maternal Age Category (years)**			
< 25	96 (24.7)	63 (20.0)	33 (44.6)
25-34	231 (59.4)	197 (62.5)	34 (45.9)
35+	62 (15.9)	55 (17.5)	7 (9.5)
Missing	0	0	0

*Women had all 3 serum cotinine measurements, both self-reported tobacco smoke interviews, meconium measurement, and infant birth outcome data.

### Relationship between Self-Report and Biomarkers of Tobacco Smoke Exposure

NIC, COT, and 3HC were detected in 80.1, 69.6, and 56.4% of infant meconium samples, respectively; 90.2% of samples had at least one detectable metabolite (Table 2). Sixty-one percent of women had mean serum cotinine concentrations > LOD and 88.9% had at least one detectable serum cotinine concentration between 16 weeks gestation and birth. Geometric mean meconium tobacco smoke metabolite concentrations were highest for NIC (GM: 2.40 ng/g; 95% CI: 2.08, 2.78) and lower for COT (GM: 0.19 ng/g; 95% CI: 0.15, 0.24) and 3HC (GM: 0.17 ng/g; 95% CI: 0.13, 0.23). Seventy-five percent of women with mean serum cotinine concentration <LOD gave birth to infants with at least one detectable meconium tobacco smoke metabolite.

**Table 2:** Descriptive statistics of meconium and serum tobacco smoke metabolite concentrations

Metabolite	N	% Detected	Minimum	**5**^**th**^	**25**^**th**^	Median	**75**^**th**^	**95**^**th**^	Maximum
Meconium NIC	331	80.1	< LOD	< LOD	1.06	2.02	4.14	48.2	301
Meconium COT	339	69.6	< LOD	< LOD	< LOD	0.12	0.40	33.5	313
Meconium HCOT	335	56.4	< LOD	< LOD	< LOD	0.10	0.52	51.2	324
Serum COT	338	61.2	< LOD	< LOD	< LOD	0.02	0.21	62.8	356

*-Meconium concentrations are ng/g and serum concentrations are ng/mL

Serum cotinine measures were highly correlated with each other (Pearson R~0.7-0.9). Meconium tobacco smoke metabolite concentrations were also highly correlated with each other: NIC and COT (Pearson R = 0.79), NIC and 3HC (Pearson R = 0.72), and COT and 3HC (Pearson R = 0.85). Mean serum cotinine and meconium metabolite concentrations were also highly correlated: NIC (Pearson R = 0.59), COT (Pearson R = 0.72), and 3HC (Pearson R = 0.71). The RSDs for the meconium tobacco smoke metabolite analyses (10-30%) were approximately 3 times higher than the RSDs for our serum metabolite analyses (3-10%).

Self-reported tobacco smoke exposures were positively associated with mean and detectable meconium tobacco smoke metabolite concentrations (Table 3). Compared to unexposed infants, women self-reporting SHS exposure gave birth to infants with slightly higher meconium nicotine concentrations and those reporting active smoking gave birth to infants with meconium nicotine concentrations 1 to 3 orders of magnitude higher. Meconium COT and 3HC concentrations were similar among women with self-report of secondhand or no tobacco smoke exposures, but higher among self-reported active smokers.

**Table 3:** Geometric mean infant meconium tobacco smoke metabolite concentration by prenatal tobacco smoke exposure.

		Meconium NIC		Meconium COT		Meconium 3HC	
	**N**	**% Detect**	**GM** **(95% CI)**	**% Detect**	**GM** **(95% CI)**	**% Detect**	**GM** **(95% CI)**

**By Self-Reported Exposure**							

Unexposed Both Periods	232	69.3	1.74 (1.50, 2.01)	60.3	0.10 (0.08, 0.12)	45.2	0.08 (0.06, 0.11)
SHS Exposure 1 Period	21	85.7	2.20 (1.35, 3.59)	95.2	0.27 (0.12, 0.62)	85.7	0.32 (0.13, 0.81)
SHS Exposure Both Periods	27	85.2	2.56 (1.67, 3.94)	77.8	0.20 (0.09, 0.40)	59.3	0.15 (0.07, 0.34)
Active Exposure 1 Period	15	86.7	4.34 (2.44, 7.73)	93.3	0.92 (0.35, 2.43)	86.7	0.91 (0.31, 2.68)
Active Exposure Both Periods	21	100	26.8 (16.6, 43.1)	100	15.9 (7.0, 36.2)	95.5	23.3 (9.5, 56.8)

**By Mean Serum Cotinine**							

Unexposed (< LOD)	131	70.6	1.50 (1.24, 1.82)	48.1	0.07 (0.05, 0.09)	33.8	0.05 (0.04, 0.07)
SHS Exposure (LOD - 3 ng/mL)	170	83.9	2.08 (1.76, 2.45)	79.4	0.16 (0.12, 0.21)	65.3	0.15 (0.12, 0.21)
Active Exposure (> 3 ng/mL)	37	94.7	21.3 (15.0, 30.1)	100	16.8 (9.7, 29.0)	97.4	21.3 (11.5, 39.3)

**By Cumulative Serum Cotinine**							

Unexposed all 3 Measures	61	71.2	1.32 (0.99, 1.76)	50	0.05 (0.03, 0.07)	30.0	0.03 (0.02, 0.05)
SHS Exposure in 1 Measure	61	65.5	1.57 (1.18, 2.10)	46.7	0.08 (0.05, 0.12)	40.0	0.07 (0.04, 0.11)
SHS Exposure in 2 Measures	41	71.8	1.70 (1.20, 2.42)	65	0.09 (0.05, 0.14)	40.0	0.07 (0.04, 0.12)
SHS Exposure in 3 Measures	92	89.2	2.41 (1.92, 3.03)	89.4	0.19 (0.14, 0.26)	72.5	0.18 (0.12, 0.27)
Active Exposure in 1 Measure	14	92.9	4.87 (2.71, 8.75)	100	2.77 (1.20, 6.37)	92.9	1.98 (0.75, 5.25)
Active Exposure in 2 Measures	3	100	24.6 (6.92, 87.3)	100	13.3 (2.20, 80.5)	100	20.6 (2.50, 170)
Active Exposure in 3 Measures	22	95.6	27.6 (17.5, 43.7)	100	20.6 (10.6, 40.0)	100	32.3 (15.1, 69.3)

Concentration in ng/g

Infant meconium tobacco smoke metabolite concentrations were greater when mother's mean serum cotinine concentrations indicated secondhand and active tobacco smoke exposure rather than no exposure (Table 3). All three meconium tobacco smoke metabolites were detected in essentially all infants born to women with mean serum cotinine concentrations > 3 ng/mL.

**Figure 1:**
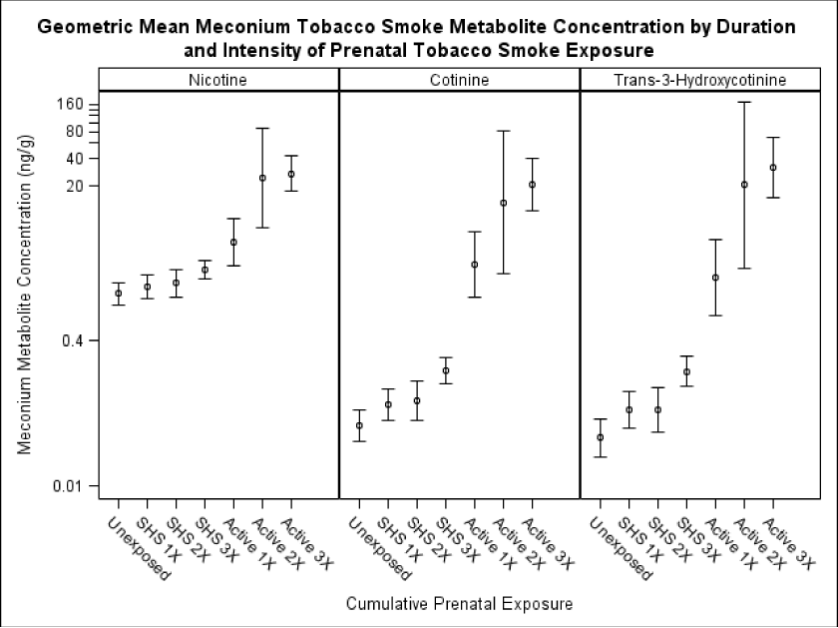
**Geometric mean meconium tobacco smoke metabolite concentration by duration and intensity of prenatal tobacco smoke exposure**. Unexposed indicates all three prenatal serum cotinine measurements were <LOD.  1X, 2X, and 3X indicate the number of serum cotinine measurements consistent with secondhand or active tobacco smoke exposure. Error bars represent 95% CI of the geometric mean.

Meconium tobacco smoke metabolite concentrations increased with the number of serum cotinine measurements indicative of secondhand or active tobacco smoke exposure (Table 3). However, there was little difference in meconium tobacco smoke metabolite concentrations among infants born to women with one or two serum measurements indicative of SHS exposure (Figure 1). Infant meconium tobacco smoke metabolite concentrations were about 2 times higher in women with three serum measurements indicative of SHS compared to women with no tobacco smoke exposure. Relative to differences in meconium NIC concentrations, meconium COT and 3HC concentrations were higher among active smokers and women with three serum measurements indicative of SHS exposure compared to women with no exposure.

**Figure 2:**
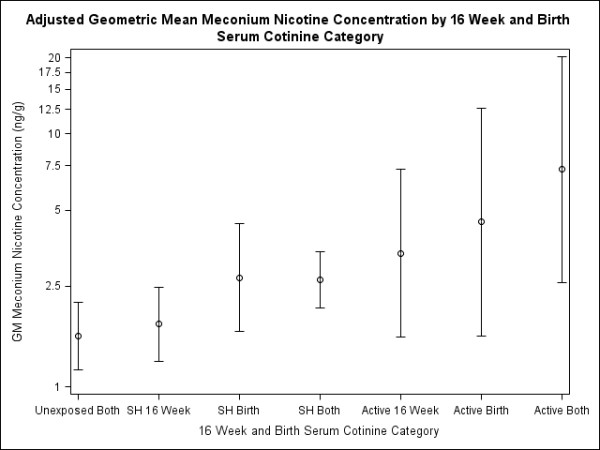
Adjusted geometric mean meconium nicotine concentration by 16 week and birth serum cotinine category*. *-Adjusted for 26 weeks serum cotinine category (< LOD, LOD - 3 ng/mL, and > 3 ng/mL).  Error bars represent 95% CI of the geometric mean.

Secondhand or active tobacco exposures in later pregnancy resulted in greater increases in meconium NIC concentrations than exposures earlier in pregnancy (Figure 2). After adjustment for 26-week serum cotinine concentrations, meconium NIC concentrations were higher among infants born to women with serum cotinine concentrations indicative of SHS exposure at birth only (GM: 2.71; 95% CI: 1.65, 4.43) compared to infants born to women with serum cotinine concentrations indicative of SHS exposure at 16 weeks only (GM: 1.77; 95% CI: 1.26, 2.48).

### Association between Biomarkers of Prenatal Tobacco Smoke Exposure and Infant Birth Weight

Of the meconium tobacco smoke metabolites, NIC provided the largest and least precise point estimates compared to meconium COT and 3HC (Table 4). Of the individual serum cotinine concentrations, birth serum cotinine categories of prenatal tobacco smoke exposure were the largest and most precise point estimates. Meconium NIC concentrations provided estimates most similar to mean prenatal serum cotinine concentrations and birth serum cotinine concentrations. Categorical meconium COT and 3HC estimates provided attenuated associations relative to birth and mean serum cotinine categories and meconium NIC categories.

**Table 4:** Adjusted association between serum and meconium biomarkers of tobacco smoke exposure and infant birth weight.*

	N	Mean Birth Weight	Change in Birth Weight (95% CI)	**Confidence Limit Difference**†
**Meconium NIC**				

Unexposed (< LOD)	62	3386	Ref	---

SHS (LOD - 10 ng/g)	212	3251	-136 (-295, 24)	320

Active (> 10 ng/g)	28	3252	-135 (-395, 126)	520

Continuous†	302	---	-62 (-178, 53)	231

**Meconium COT**				

Unexposed (< LOD)	97	3317	Ref	---

SHS (LOD - 5 ng/g)	171	3286	-27 (-175, 120)	295

Active (> 5 ng/g)	34	3207	-106 (-348, 135)	482

Continuous†	302	---	-61 (-132, 10)	143

**Meconium 3HC**				

Unexposed (< LOD)	135	3335	Ref	---

SHS (LOD - 10 ng/g)	139	3235	-100 (-246, 46)	292

Active (> 10 ng/g)	28	3278	-57 (-307, 192)	500

Continuous†	302	---	-30 (-96, 36)	132

**Mean Serum COT**				

Unexposed (< LOD)	122	3377	Reference	---

SHS (LOD to 3 ng/mL)	148	3265	-112 (-264, 41)	305

Active (> 3 ng/mL)	32	3188	-189 (-462, 84)	545

Continuous†	302	---	-60 (-135, 16)	150

**16 Week Serum COT**				

Unexposed (< LOD)	101	3312	Reference	---

SHS (LOD to 3 ng/mL)	162	3292	-20 (-173, 132)	305

Active (> 3 ng/mL)	32	3203	-109 (-386, 168)	554

Continuous†	295	---	-59 (-125, 7)	132

**26 Week Serum COT**				

Unexposed (< LOD)	126	3282	Reference	---

SHS (LOD to 3 ng/mL)	138	3302	20 (-135, 175)	305

Active (> 3 ng/mL)	29	3144	-138 (-425, 150)	554

Continuous†	293	---	-41 (-104, 21)	125

**Birth Serum COT**				

Unexposed (< LOD)	121	3416	Reference	---

SHS (LOD to 3 ng/mL)	131	3319	-97 (-248, 54)	302

Active (> 3 ng/mL)	29	3226	-190 (-462, 82)	545

Continuous†	281	---	-40 (-104, 24)	128

*-Adjusted for maternal age (18 to <25, 25 to <35, and 35+ years), race (non-Hispanic white, non-Hispanic black, and other), education (< 12, 12, and >12 years), martial status (married and unmarried), depression (minimal, mild, and moderate/severe), parity (0, 1, and >1) and maternal weight.

†-Calculated by subtracting the lower 95% confidence limit from the upper 95% confidence limit

‡-Coefficients represent change in birth weight for a 10-fold increase in metabolite concentration.

Estimates between SHS exposure and birth weight were similar when we evaluated different thresholds for classifying SHS and active exposure using meconium tobacco smoke metabolite concentrations. However, raising the threshold for active exposure (10 to 30 ng/g) increased NIC point estimates between active smoking and birth weight and raising the threshold for COT (5 to 10 ng/g) and 3HC (10 to 30 ng/g) decreased the point estimate between active smoking and birth weight.

## Discussion

The two sets of analyses presented suggest that meconium can be used as a biological matrix to measure prenatal tobacco smoke exposure. Meconium tobacco smoke metabolites were positively associated with self-report and serum biomarkers of prenatal tobacco smoke exposure. We observed a dose-dependent relationship between the number of serum cotinine measurements consistent with secondhand or active tobacco smoke exposure during the latter two-thirds of pregnancy and meconium tobacco smoke metabolite concentrations. Our results indicate that tobacco smoke metabolites in meconium reflect the duration and intensity of gestational exposure to tobacco smoke. Tobacco smoke metabolites may accumulate in meconium differentially across pregnancy since the bulk of meconium is formed later in pregnancy.

Meconium COT and 3HC concentrations were higher and almost universally detected among infants born to active smokers compared to women with secondhand or no exposure. Meconium COT and 3HC concentrations may be a more sensitive biomarker of active prenatal tobacco smoke exposures, while meconium NIC concentrations may be a more sensitive marker of secondhand exposures since they were detected more frequently among infants with secondhand or no tobacco smoke exposure. An additional advantage to meconium NIC is that the higher frequency of detection reduces the need to impute left-censored data [21].

Meconium may be a more sensitive matrix to measure prenatal tobacco smoke exposure than serum if it reflects transient exposures that may not be captured by individual or serial serum measurements. However, non-detectable serum cotinine concentrations could be due to increased nicotine and cotinine metabolism and clearance during pregnancy [13,31]. Thus, women with tobacco smoke exposures that are not detectable using serum cotinine might give birth to infants with detectable meconium tobacco smoke metabolites. We were not able to examine whether metabolic or genetic factors, like CYP2A6 enzyme activity, modified the relationship between tobacco smoke exposures and meconium tobacco smoke metabolites [32,33].

We detected a higher proportion of some meconium tobacco smoke metabolites than some previous studies [9,11]. Our proportion of detectable meconium COT and 3HC was similar to Gray and colleagues [12]. Another study reported almost universal meconium COT detection among their study participants [16]. Variations in study results could be due to differences in meconium digestion/extraction, analytical chemistry methods, or exposure characteristics of the targeted study population.

Tobacco smoke exposures in later pregnancy may cause greater increases in meconium tobacco smoke metabolite concentrations relative to earlier exposures. This complicates the interpretation of meconium metabolite concentrations since they reflect the duration, intensity, *and *timing of exposure. Differential accumulation of tobacco smoke metabolites in meconium over the course of pregnancy may be due to changes in blood volume, kidney and liver metabolism, placental perfusion, increased quantities of amniotic fluid ingested by the infant later in gestation, or amount of meconium formed in later gestation [34]. However, inferences regarding the timing of tobacco smoke exposures are based on a relatively small number of women and infants with different temporal patterns of exposure.

We are not aware of previous studies that attempted to validate meconium as a matrix for measuring biomarkers of prenatal tobacco smoke exposure using repeated serum cotinine measures for comparison. Ostrea et al. reported that meconium nicotine concentrations increased with self-reported prenatal tobacco smoke exposure intensity [3]. Kohler et al. reported higher meconium NIC, COT, and 3HC concentrations among women with greater duration of active smoking during pregnancy compared to women who quit smoking earlier in pregnancy, but these results were based on only eleven women who quit smoking during pregnancy [11]. Serial serum cotinine measurements allowed us to more accurately classify prenatal secondhand and active tobacco smoke exposures than previous studies.

Both serum cotinine and meconium tobacco smoke metabolite concentrations were inversely associated with birth weight. The magnitude and precision of the point estimates using meconium NIC concentrations was similar to serum cotinine concentration estimates within our cohort and to previous estimates of the association between prenatal serum cotinine concentrations and infant birth weight [23-27]. Categorical meconium COT and 3HC point estimates were smaller in magnitude relative to categorical meconium NIC and serum cotinine point estimates; however, point estimates using continuous meconium tobacco smoke metabolite or serum cotinine concentrations were very similar to one another. Investigators may wish to use serum cotinine measurements to quantify prenatal exposure since collecting meconium samples will require hospital staff be able and willing to properly collect and store meconium samples.

There are some limitations to the presented results. First, we considered mean prenatal serum cotinine concentrations as the gold standard for prenatal tobacco smoke exposure in these analyses. Serum cotinine concentrations are a reasonable choice to compare a new biomarker of tobacco smoke exposure against since they are a more sensitive marker of secondhand exposure than self-report during pregnancy [35-37]. However, it would have been ideal to compare meconium to another long term biomarker of prenatal tobacco smoke exposure like hair nicotine or cotinine.

The small number of actively smoking women in our sample limited our ability to precisely estimate relationships between prenatal tobacco smoke exposure and meconium tobacco smoke metabolites among active smokers. However, among the larger number of women with SHS exposure, we did observe similar patterns of association between the number serum cotinine measurements indicative of SHS exposure and meconium tobacco smoke metabolite concentrations.

Finally, women in our sample were from relatively high socioeconomic background, which is associated with decreased active and SHS exposure during pregnancy [35,36,38]. Thus, our results may not be generalizable to samples from populations with lower socioeconomic status who may have different exposure distributions.

There are several advantages and disadvantages to using meconium as a matrix to measure prenatal tobacco smoke exposure in epidemiological studies. First, meconium tobacco smoke metabolite concentrations reflect the duration and intensity of prenatal exposures, providing an accurate estimate of the dose of tobacco smoke constituents received by the infant in the latter part of pregnancy. In addition, meconium may be a good matrix to measure transient prenatal tobacco smoke exposures, as we detected nicotine in the meconium of infants born to women with undetectable serum cotinine concentrations and single serum cotinine measurement indicative of secondhand or active tobacco smoke exposure. However, meconium tobacco smoke metabolite concentrations do not allow classification of exposure during specific time periods of development. Furthermore, elevated meconium tobacco smoke metabolite concentrations may be due to constant exposure over the entire course of pregnancy or high exposure in the latter parts of pregnancy.

Second, meconium could be used as a matrix for biomarkers of exposure in research studies that enroll women at or shortly after parturition, but does require additional resources to collect and analyze. While both serum and meconium biomarkers provided similar estimates of association with birth weight, investigators should consider the additional resources necessary to collect and analyze meconium samples, especially if there are other well-developed biomarkers of exposure. Meconium digestion and analysis, done at the CDC laboratory, was more labor intensive and less efficient than serum cotinine assays, taking approximately 4 times longer to complete.

## Conclusions

Meconium tobacco smoke metabolites can be used to measure the duration and intensity of prenatal tobacco smoke exposure. However, investigators planning to use meconium should consider whether meconium toxicant concentrations will provide additional exposure information not gleaned from other well-developed biomarkers of exposure. For studies of prenatal tobacco smoke exposure, meconium tobacco smoke metabolites do not provide additional exposure information that would not be captured by a single serum cotinine measurement. Additional research should determine whether meconium can be used estimate gestational exposure to other environmental toxicants that exhibit more variability during pregnancy, especially non-persistent compounds like bisphenol A and phthalates. Future studies should compare meconium and other validated biomarkers of exposure with infant or child health outcomes in well characterized exposure-outcome relations.

## List of Abbreviations

BDI-II: Beck Depression Inventory II; CDC: Centers for Disease Control and Prevention; CI: Confidence Interval; CLD: Confidence Limit Difference; COT: Cotinine; CV: Coefficient of Variation; DAG: Directed Acyclic Graph; GM: Geometric Mean; HCOT: Trans-3'-hyrdoxycotinine; HOME: Health Outcomes and Measures of the Environment; IRB: Institutional Review Board; KG: Kilogram; LOD: Limit of Detection; NIC: Nicotine; NG/ML: nanograms per milliliter; NG/G: nanograms per gram; QC: Quality Control; SHS: Secondhand Tobacco Smoke.

## Competing Financial interests

The authors declare that they have no competing interests.

## Authors' contributions

JMB reviewed the literature, analyzed the data, and drafted the manuscript. JLD, CP, AFO, and RH provided feedback and guidance on data analysis and manuscript preparation. JTB developed the meconium tobacco smoke metabolite assay and provided feedback on the manuscript. YX helped in developing the meconium tobacco smoke metabolite assays. CB and DBB provided feedback on the manuscript and planned the meconium collection and analysis procedures for the HOME study. BPL conceived the original study idea and provided feedback on data analysis and manuscript drafts. All authors read and approved the final manuscript.

## Disclaimer

The findings and conclusions in this report are those of the author(s) and do not necessarily represent the official position of the Centers for Disease Control and Prevention.
